# Cost-effectiveness analysis of including contrast-enhanced ultrasound in management of pancreatic cystic neoplasms

**DOI:** 10.1007/s11547-022-01459-8

**Published:** 2022-03-01

**Authors:** Niccolo’ Faccioli, Elena Santi, Giovanni Foti, Mirko D’Onofrio

**Affiliations:** 1 Present Address: Department of Radiology, G.B. Rossi Hospital, Università di Verona, Piazzale L.A.Scuro 10, 37134 Verona, Italy; 2 IRCCS Sacro Cuore Don Calabria Hospital, Negrar, Verona, Italy

**Keywords:** Economic evaluation, Incidental pancreatic lesion, Optimal strategy, Quality of life, Surveillance

## Abstract

**Purpose:**

Pancreatic cystic neoplasms (PCN) management consists of non-invasive imaging studies (CT, MRI), with a high resource burden. We aimed to determine the cost-effectiveness of including contrast-enhanced ultrasound (CEUS) in the management of PCN without risk features.

**Materials and methods:**

By using a decision-tree model in a hypothetical cohort of patients, we compared management strategy including CEUS with the latest Fukuoka consensus, European and Italian guidelines. Our strategy for BD-IPMN/MCN < 1 cm includes 1 CEUS annually. For those between 1 and 2 cm, it includes CEUS 4 times/year during the first year, then 3 times/year for 4 years and then annually. For those between 2 and 3 cm, it comprises MRI twice/year during the first one, then alternating 2 CEUS and 1 MRI yearly.

**Results:**

CEUS surveillance is the dominant strategy in all scenarios. CEUS surveillance average cost is 1,984.72 €, mean QALY 11.79 and mean ICER 181.99 €. If willingness to pay is 30,000 €, 45% of patients undergone CEUS surveillance of BDIPMN/MCN < 1 cm would be within budget.

**Conclusion:**

Guidelines strategies are very effective, but costs are relatively high from a policy perspective. CEUS surveillance may be a cost-effective strategy yielding a nearly high QALYs, an acceptable ICER, and a lower cost.

## Introduction

Pancreatic cystic neoplasms (PCN) are closed cavities, usually containing liquid or mucinous material; their prevalence in asymptomatic individuals is estimated to be 8% [1], and represent a heterogeneous group of tumours, each of them with typical biological behaviour. Over 90% of incidental PCN can be categorized as serous cystic neoplasm (SCN), intraductal papillary neoplasm (IPMN) or mucinous cystic neoplasm (MCN). The premalignant risk of PCNs varies according to the type of lesion, size, and histological subtype [2]. SCN represents 10–16% of cystic pancreatic neoplasms, they are benign in nearly all the cases, and should be followed up yearly. Indications for surgery are symptoms and increasing tumour diameter [3–5]. IPMN represents the most common PCN, and can be classified as main duct type, mixed type, and branch duct type [4–7]. Branch-duct IPMNs (BD-IPMNs) have a less clear indication for surgery, as the rate of pancreatic invasive malignancy (2%–3.7%) is comparable to the risk of mortality following pancreatectomy [6–9]. In the 2017 revised version of the international guidelines for the management of BD-IPMN [10], worrisome features (cyst size > 3 cm, main pancreatic duct size of 5–9 mm, pancreatitis, non-enhancing nodules, thickened and enhanced cyst wall, main duct stricture with upstream dilatation, and peripancreatic lymphadenopathy) and high-risk stigmata (jaundice, MPD ≥ 10 mm and enhancing nodules) are described as indications to better analyse the morphology of these lesions, and to stratify the risk of malignancy. Surveillance (cross-sectional imaging in lesions < 20 mm, EUS or MRI in lesions > 20 mm) is proposed for BD-IPMN without high-risk stigmata, with the time interval depending on the size of the lesion [10]. MCN are associated with a potential risk to develop malignancy in less than 20% of cases [1, 2]. For MCN measuring < 40 mm without a mural nodule or symptoms, surveillance with MRI, EUS or a combination of both is recommended [11–13]. The clinical-radiological surveillance of PCNs has become a challenge for health systems considering their costs and resource burden [7, 8, 14]. In routine clinical practice, cystic lesions management consists on non-invasive imaging studies (CT, MRI) according to the last recommendations [10–13]; and by more invasive tests such as Endoscopic US (EUS) [14]. Cystic lesions management is therefore a significant health issue, not solely from a diagnostic point of view, but also for its costs. In recent years, contrast-enhanced ultrasound (CEUS) has become more important in the evaluation of pancreatic neoplasms, if previously visible on ultrasound [15–17]. Some studies have shown that diagnostic accuracy of CEUS is analogous to MRI in the detection of septa and mural nodules of PCNs and can reveal vegetations’ enhancement [15–21]. In 2011, EFSUMB guidelines included CEUS in pancreatic evaluation, especially with respect to discriminating solid and cystic lesions [21].

Keeping in mind the latest Guidelines [10–13, 21], we simulate alternative follow-up algorithms for cystic pancreatic lesions in which CEUS could play a key role and could represent a cost-effective imaging method, for a high quality but cheaper healthcare [22, 23]. The purpose of this paper is to determine the cost-effectiveness of inclusion of CEUS in the management of asymptomatic BD-IPMNs < 3 cm without worrisome features and SCNs/MCNs < 4 cm without risk of malignant progression. The outcome could have a large economic impact on current practice due to the difference in cost of the imaging modalities.

## Materials and methods

By using an analysis software (OpenMarkov™; CISIAD, UNED, Madrid, Spain), we conducted an economic-based simulation study using a linear decision tree, to compare surveillance using Consensus Guidelines and surveillance with the inclusion of CEUS for a hypothetical cohort of 1000 patients with a variety of asymptomatic pancreatic cysts ranging from 0.5 to 4 cm in the head of the pancreas, without any worrisome features or risk of malignant progression.

**Fig. 1:**
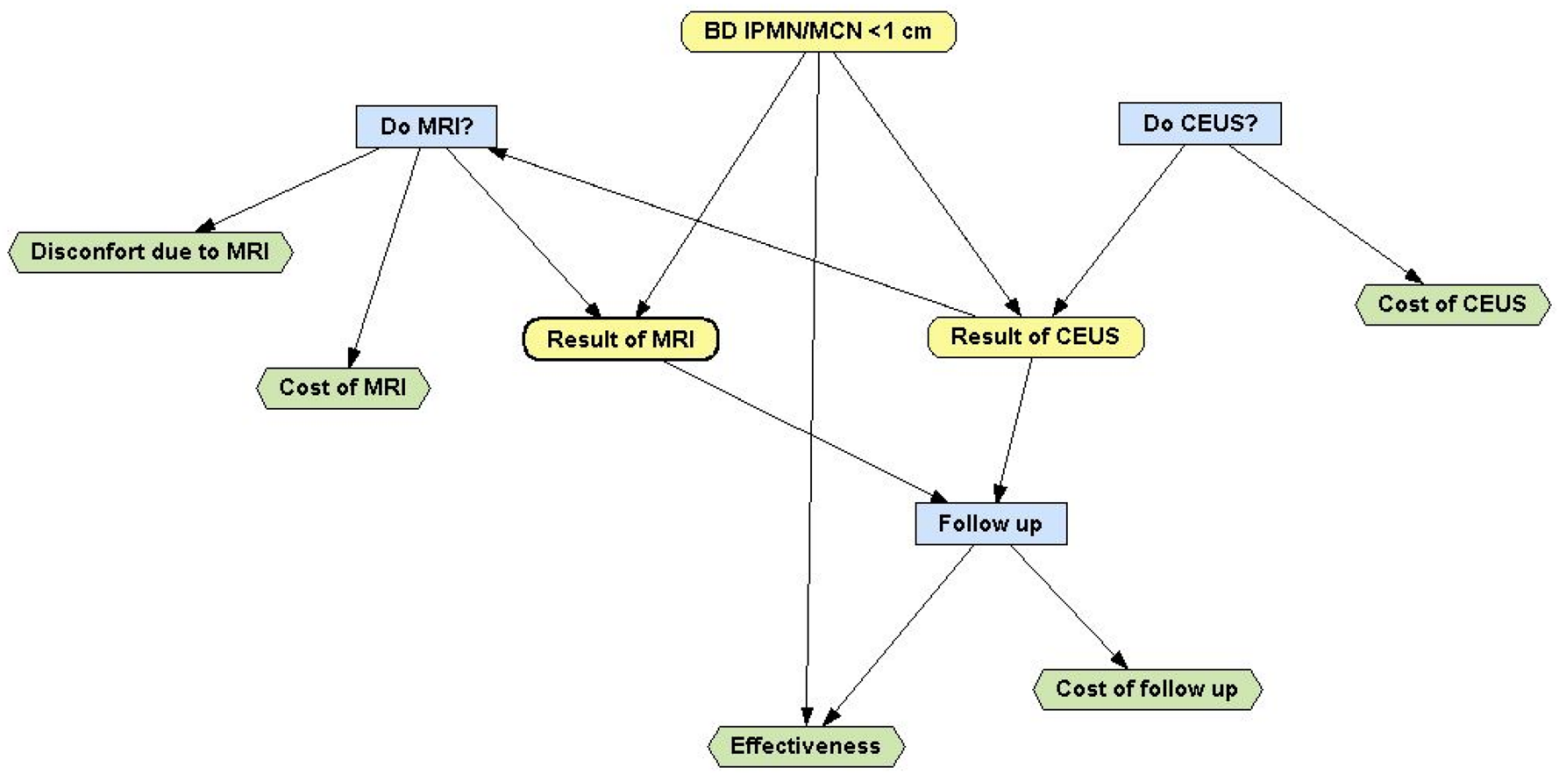
Example of decisional tree for management strategies in BD-IPMN/MCN < 1 cm

We chose a decision tree as a one-period model in which branches represent chains of possible events, each with a certain probability of occurrence (Fig. 1). The natural history of patients with PCN is modelled by using three health states associated with asymptomatic PCN: healthy, sick, and deceased in the Markov model, related to a set of costs and utilities. Transitions between health states were permitted at the end of each model cycle, which was set to 10 years [24, 25]. Cohort members were redistributed to different health states depending on the estimated probabilities of transition. We assumed that the survival of patients with benign pancreatic cysts is the same as the age-specific population from European Life Table [26–28]. If a patient has no changes in follow-up, the pathway reaches a terminal node. Health state transitions are defined by the event probabilities that are summarized in Table 1. All model variable estimates were derived from data reported in literature and are summarized in Table 1. OpenMarkov™ (CISIAD, UNED, Madrid, Spain), allows the user to impose policies on decision nodes: the purpose of imposing policies is to analyse the behaviour of all scenarios, also for those that never occur if the decision maker applies the optimal strategy [24, 25, 29].

**Table 1:** List of parameters used in Markov model

Parameters in the model	Baseline estimate	Reference no
Total number of cycles	10	
*Base case*		
Correct diagnosis	0.85	1,5,7,34
Probability to have a benign pancreatic cyst	0.10	1–3,5–7
Probability to have a malignant pancreatic cyst	0.05	1–3,5,7,20,31
Location in head or neck of the pancreas	50%	1,3,7
Location in body or tail of the pancreas	50%	1,3,7
Proportion of mucinous cystic lesion/branch-type IPMN at presentation	0.65	1,2,7,9,20
Proportion of non-mucinous cystic lesions	0.3	1,3,7
Annual probability of cystic lesion transitioning from asymptomatic to symptomatic state	0.02	6,8,31–33
Probability that a benign cyst grows	0.05	6,8,31–33
Probability of dying from an EUS-FNA	0.0001	18
Probability of dying from a malignant IPMN without treatment	0.6	6,7,20
*Mortality*		
Background mortality	Age specific	28
*Annual Costs*		
Abdominopelvic CT	106.23 €	30
Abdominal MRI	219.61 €	30
Endoscopic ultrasonography	739 €	30
CEUS	70.50 €	30
*Utility values of cross-sectional imaging*		
CEUS sensitivity	79–94%	15–17,19,22–23,36
CEUS specificity	76–99%	15–17,19,22–23,36
CT sensitivity	57–69%	34,37
CT specificity	63–83%	34,37
MRI sensitivity	75–82%	15,37
MRI specificity	94–96%	15,37
EUS sensitivity	78–83%	18–19
EUS specificity	91–95%	18–19
*Health utility*		
Base-case	0.80	
Annual decrease (aging)	− 0.01	28
Instant decrease (Symptoms)	− 0.03	41
Quality of life (utility) of undergoing invasive surveillance	0.73	38–39,41
Quality of life (utility) of undergoing non-invasive surveillance	0.78	38–39,41
Quality of life (utility) of developing malignant pancreatic cyst	0.68	38–39,41

Table 1 lists all parameters of the model. We analysed our model from a societal perspective that included direct costs for CEUS, CT, MRI and EUS as cost of equipment and its maintenance, cost of materials (contrast material, needles and injections devices, archiving supports) and human resources, provided by the Hospital Technical Department [30]. Total costs were: 70.50 € for abdominal contrast-enhanced ultrasound; 106.23 € for CT; 219.61 € for upper abdominal MRI and 739 € for EUS. Based on the literature and on our experience in a high-volume centre for pancreatic pathologies, we estimated that 15% of the initial cohort would have malignant branch IPMN, of which 59% and 41% would be carcinoma in situ (CIS) and invasive cancer, respectively [6, 31–35]. Performance characteristics and utility values of cross-sectional imaging studies and EUS were derived from published information and summarized in Table 1 [36, 37].

The primary outcomes compared among the strategies were QALYs (quality adjusted life years), ICER (incremental cost-effectiveness ratio) and NMB (net monetary benefit). To calculate the total QALYs for each diagnostic strategy, we incorporated a range of health-related quality-of-life estimates based on previously published data regarding asymptomatic PCN [38, 39]. We based health related quality of life on studies where generic QoL is measured by the EuroQol (EQ-5D). The European Organization for Research and Treatment of Cancer (EORTC) QOL Questionnaire C30 (QLQ-C30) and its pancreas module (QLQ-PAN26) were used as generic and disease-specific QOL instruments [40, 41]. These utility instruments in the form of a patient-reported questionnaire represent a single health state by documenting several domains, each ranked on a scale of 1–3, representing none, some, or extreme problems in that area. We compared surveillance using Consensus Guidelines and surveillance with the inclusion of CEUS for a hypothetical cohort of 1000 patients with a variety of asymptomatic pancreatic cysts ranging from 0.5 to 4 cm in the head of the pancreas, without any worrisome features or any risk of malignant progression. We considered the baseline scenario of a 60-year-old patient found at MRI to have an asymptomatic solitary BD-IPMN without any worrisome features or high-risk stigmata. Although the base-case patient was considered to have likely BD-IPMN based on typical clinical features, we ensured that the patient was eligible to have other cystic lesions. For BD-IPMN, gender ratio was assumed to be 1:1; for MCN 9:1 (female: male) and for SCN 4:1 (female: male). The time horizon of the models is 10 years. To test a variety of pancreatic neoplasm size, we ran separate models to estimate outcomes in BD-IPMN (< 1 cm, 1–2 cm, 2–3 cm) and MCN/SCN < 4 cm. Risk of misdiagnosis was included both for a benign lesion and for malignant disease [34, 37]. Age-specific all-cause mortality, based on data from European Life Table [28], was added to simulate death from other causes.

This study is performed from the health care sector perspective and we consider only direct costs of diagnostic tests. International Consensus Fukuoka guidelines for the management of BD-IPMN < 1 cm cystic lesion include CT/MRI in 6 months, then every 2 years if no change [10]. Italian consensus guidelines’ strategy for BD-IPMN with diameter < 1 cm, visible at US, is US every 12 months until size change occurs; then, CEUS or MRI imaging every 12 months should be performed to evaluate the presence of high-risk features (size, nodules, septa, content, morphology). MRI with MRCP, alternated with US, should be used to evaluate the development of new PCNs. If MRI identifies new PCNs, a follow-up must be carried out with MRI. If after two years from initial diagnosis the branch duct IPMN is stable, imaging will be every 24 months [11]. Our strategy is based on surveillance with CEUS annually. Consensus Fukuoka guidelines’ strategy for BD-IPMN between 1–2 cm foresees CT/MRI every 6 months for 1 year, yearly for 2 years, then lengthen interval up to 2 years if no change [10]. Italian consensus guidelines’ strategy for BD-IPMN with diameter 1–2 cm visible in the US every 6–12 months is preferred until size change occurs. If size change occurs, CEUS or MRI imaging should be performed. If not visible in the US: MRI with MRCP or MDCT. If after two years from initial diagnosis the branch duct IPMN is stable, imaging will be every 18 months [11]. Our strategy is based on surveillance with CEUS every 3 months for the first year, then every 4 months for 4 years and then annually for 5 years if stable. Consensus Fukuoka guidelines’ strategy for BD-IPMN between 2–3 cm comprises EUS in 3–6 months, then lengthen interval up to 1-year, alternating MRI with EUS as appropriate [10]. Italian consensus guidelines’ strategy for IPMN with diameter greater than 20 mm is MRI with MRCP or MDCT every 3–6 months. If after two years from initial diagnosis the branch duct IPMN is stable, follow-up timing can be modified as follows: MRI with MRCP or MDCT every 12 months [11]. Our strategy is based on surveillance with MRI every 6 months for the first year, then close surveillance alternating 2 CEUS and 1 MRI every year for 9 years. European Guidelines’ strategy for MCN measuring < 30 mm without a mural nodule or symptoms is comparable to BD-IPMN follow-up strategies [13]. In case of MCN measuring 30–40 mm, surveillance consists of MRI, EUS, or a combination of both, every 6 months for 3 years, then annually if no changes are observed [12]. MCN ≥ 40 mm should undergo surgical resection. Resection is also recommended for MCN which are symptomatic or have risk factors, irrespective of their size [11–13]. Our strategy is based on surveillance with MRI every 3 months for the first year, then close surveillance with 3 CEUS and 1 MRI every year for 9 years. European Guidelines’ strategy for asymptomatic patients with SCN is follow-up with MRI for 1 year. After 1 year, symptom-based follow-up is recommended. Only when the diagnosis is uncertain is follow-up required. In these cases, a patient should undergo the same follow-up as for a BD-IPMN [13]. Italian consensus guidelines’ follow-up is on a yearly basis: cyst size should be evaluated over time and US could therefore be used. If there is cyst growth and/or the presence of symptoms, then MRI with MRCP or CT should be performed. If no growth occurs for 3 years, consider stopping the follow-up [11]. Our strategy is based on surveillance with CEUS yearly for 3 years. In case of suspicious features, MRI with MRCP alternate with CEUS should be carried out, undergoing the same follow-up as for a BD-IPMN. If the cystic lesions represented a benign clinical course, staying asymptomatic during follow-up with no risk of malignant transformation, CEUS surveillance was performed in these patients for all 10 years. If the lesion became symptomatic or showed significant growth on follow-up with a risk of malignant transformation, the patient would require closer surveillance with MRI or EUS-FNA. If suspicious or positive for malignancy, surgery is strongly recommended [10–14, 31–35].

We based our probabilistic sensitivity analysis on the second-order distributions assigned to some parameters, carried out by means of stochastic simulations (Monte Carlo techniques) [24, 25]. Then, we performed one-way sensitivity analysis (tornado diagrams) and probabilistic cost-effectiveness sensitivity analysis (acceptability curves) for all variables with a specified range, as shown in Table 1 to determine the thresholds where the most cost-effective strategy would change. Using a 30,000€ willingness-to-pay (WTP) threshold, finally we obtained an estimate of the probability of each follow-up being optimal for the value of WTP itself. Probabilistic sensitivity analyses were performed to test the result of uncertainty for costs and effects. The gamma distribution was designated for cost parameters, and the normal distribution was selected for probability, proportion and quality of life value parameters.

## Results

**Fig. 2:**
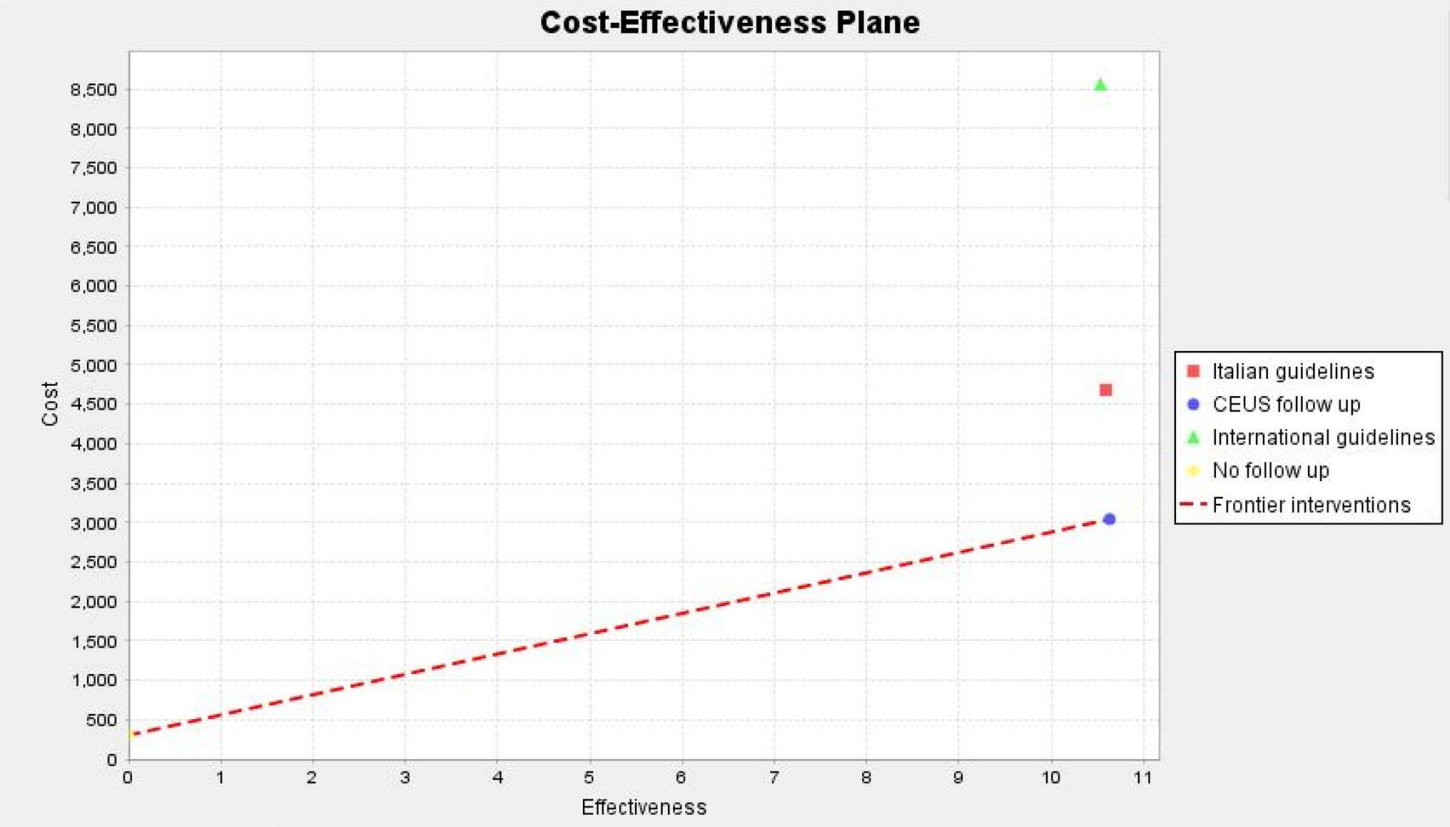
Plot of cost versus effectiveness for management strategies in BD-IPMN 2–3 cm. The horizontal axis represents the effectiveness and the vertical one the cost. The slope of line that connects the points is determined by their ICER (frontier interventions). Willingness to pay (WTP) set to 30,000€

The results of the base-case analyses are displayed on Table 2, stratified by pancreatic cyst type and size. Consensus Fukuoka Guidelines’ surveillance average cost, calculated for BD-IPMN, is 5102.4 €; mean QALY is 10.89, mean ICER is 505.05 € and mean NMB is 10.727. Consensus Italian Guidelines’ surveillance average cost, calculated for BD-IPMN < 3 cm and SCN, is 2136.7 €; mean QALY is 11.73, mean ICER is 190.84 € and mean NMB is 11.65. CEUS surveillance average cost, calculated for all cyst categories, is 1984.72 €, mean QALY 11.79, mean ICER is 181.93 € and mean NMB is 11.74. For all cyst categories, follow-up CEUS strategy is both clinically superior and cost saving, resulting in an economically “dominant” strategy. We can observe, for example, the plot in the cost-effectiveness plane tab for BD-IPMN 2–3 cm scenario (Fig. 2) where “CEUS follow-up” is both cost-saving and clinically beneficial.

**Table 2:** Results of Probabilistic Sensitivity Analysis for each management strategies

BD-IPMN/MCN < 1 cm	Cost (Euro)	Effectiveness (QALY)	ICER (Euro/QALY)	NMB	iNMB
Consensus Fukuoka guidelines	1537.27	12.54	121.63	12.48	1.05
Italian consensus guidelines	828.22	13.37	61.04	13.34	0.2
CEUS follow-up	705	13.57	51.06	13.54	/
BD-IPMN/MCN 1–2 cm	Cost	Effectiveness (QALY)	ICER (Euro/QALY)	NMB	iNMB
Consensus Fukuoka guidelines	1756.88	11.32	154.14	11.26	0.2
Italian consensus guidelines	1663.95	11.46	144.15	11.40	0.1
CEUS follow-up	1480.5	11.59	126.70	11.54	/
BD-IPMN/MCN 2–3 cm	Cost	Effectiveness (QALY)	ICER (Euro/QALY)	NMB	iNMB
Consensus Fukuoka guidelines	8268.44	10.53	784.09	10.25	0.2
Italian consensus guidelines	4392.2	10.59	413.62	10.44	0.09
CEUS follow-up	2751.99	10.63	257.76	10.53	/
MCN 3–4 cm	Cost	Effectiveness (QALY)	ICER (Euro/QALY)	NMB	iNMB
ACG Clinical Guideline and European evidence-based guidelines	8847.1	9.2	960.34	8.9	0.6
CEUS follow-up	3684.71	9.7	378.62	9.5	/
SCN < 4 cm	Cost	Effectiveness (QALY)	ICER (Euro/QALY)	NMB	iNMB
European evidence-based guidelines	2196.1	12.59	173.48	12.51	0.89
Italian consensus guidelines	1662.4	13.4	124.06	13.34	0.072
CEUS follow-up	1301.44	13.46	95.79	13.41	/

*QALY* quality-adjusted life-years, *ICER* incremental cost-effectiveness ratio. *NMB* net monetary benefit. *iNMB* incremental net monetary benefit

Tornado diagrams are useful as deterministic sensitivity analysis tools comparing the relative importance of variables. In all scenarios, the first assumption “effectiveness of CEUS follow-up” has the highest sensitivity ranking and is the most important. The last assumptions, “cost of CEUS, International and Italian guidelines”, are the least influential assumptions. Consequently, an increase in any of the costs leads to a decrease of the expected utility while a decrease in the effectiveness, sensitivity or in the specificity of the tests make the expected utility increase.

**Fig. 3:**
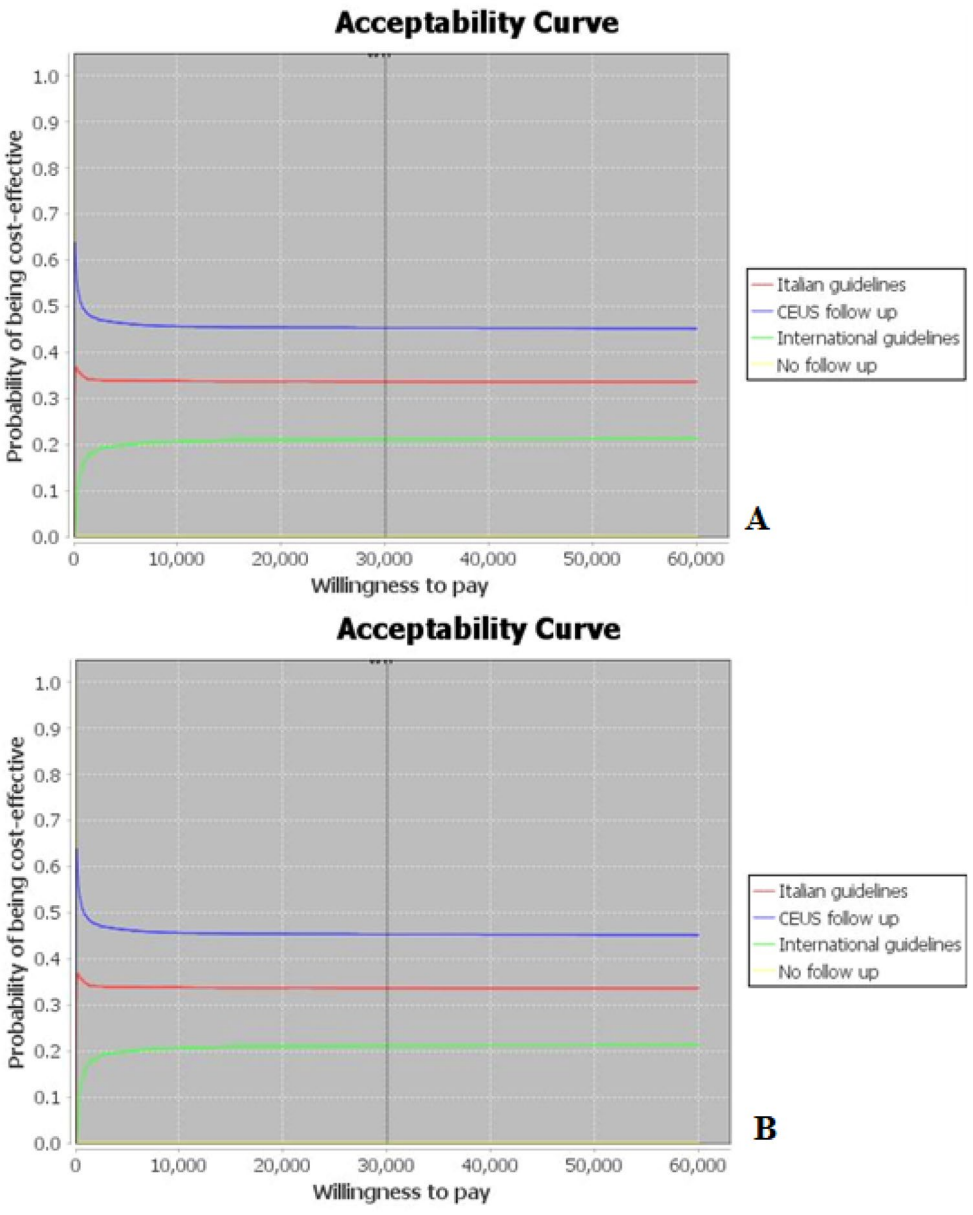
**A**. Acceptability curve for management strategies in BD-IPMN/MCN < 1 cm. At € 30,000 willingness-to-pay (WTP) threshold, 45% of the simulated trials in the surveillance strategy were within budget. **B**. Acceptability curve for management strategies in MCN 3–4 cm. At € 30,000 willingness-to-pay (WTP) threshold, 56% of the simulated trials in the surveillance strategy were within budget

The acceptability curve built in the scenario with BD-IPMN/MCN size < 1 cm is shown in Fig. 3A. When WTP is 30,000€/QALY, “CEUS follow-up “is more cost-effective than “Italian and International follow-up” and 45% of the simulated trials in the surveillance strategy are within budget. In case of “Italian guidelines follow-up” and “International guidelines follow-up”, respectively 34% and 20% of the simulated trials in the surveillance strategy are within budget. We can observe that the option “follow-up CEUS” is cheaper and more effective than management proposed in Consensus Fukuoka guidelines and Italian consensus guidelines. Incremental NMB between “follow-up CEUS” and “Consensus Fukuoka guidelines follow-up” is 1.05 with a saving of 54.13% (Table 3); between “follow-up CEUS” and “Italian consensus guidelines follow-up” is 0.2 with a saving of 14.87%. The acceptability curves built in the scenario with BD-IPMN/MCN size 1–2 cm shows that, for a WTP of €30,000/QALY, there is 36% probability of “CEUS follow-up “being the optimal treatment; the probability of “Italian consensus follow-up” being optimal is 34% and probability of “Consensus Fukuoka guidelines follow-up” is 30%. The options “follow-up CEUS”, “Consensus Fukuoka guidelines follow-up” and “Italian consensus follow-up” are very nearly effective; incremental NMB are 0.2 and 0.1, with savings of 15.7% and 11.02%, respectively. The acceptability curves built in the scenario with BD-IPMN/MCN size 2–3 cm shows that, for a WTP of 30,000€/QALY there is 40% probability of “CEUS follow-up”, being the optimal treatment. The options “follow-up CEUS”, “Consensus Fukuoka guidelines follow-up” and “Italian consensus follow-up” are very nearly effective: incremental NMB are 0.2 and 0.09, with savings of 66.7% and 37.3%, respectively. The acceptability curve in the scenario with MCN size 3–4 cm is shown in Fig. 3B: when WTP is 30,000€/QALY, the optimal intervention is to apply follow-up with CEUS and 56% of the simulated trials in the surveillance strategy are within budget. Incremental NMB between management proposed by ACG Clinical Guideline and European evidence-based guidelines and “CEUS follow-up” is 0.6. Saving amounts to 58.35%. The acceptability curve in the scenario with SCN size < 4 cm shows that WTP is 30,000€/QALY, the optimal intervention is to apply follow-up with CEUS and 41% of the simulated trials in the surveillance strategy are within budget. The option “follow-up CEUS” is the dominant strategy. Incremental NMB between management proposed by European evidence-based guidelines, Italian consensus guidelines and “CEUS follow-up” is 0.89 and 0.072, with savings of 40.73% and 19.78%, respectively.

**Table 3:** Total savings with CEUS follow-up respect to traditional Guidelines’ follow-up

BD-IPMN/MCN < 1 cm	Fukuoka guidelines follow-up vs CEUS follow-up	Italian guidelines follow-up vs CEUS follow-up
Saving (Euro)	832.27	12.22
Percentual saving	54.13%	14.87%

BD-IPMN/MCN 1–2 cm	Fukuoka guidelines follow-up vs CEUS follow-up	Italian guidelines follow-up vs CEUS follow-up
Saving (Euro)	276.38	183.45
Percentual saving	15.73%	11.02%

BD-IPMN/MCN 2–3 cm	Fukuoka guidelines follow-up vs CEUS follow-up	Italian guidelines follow-up vs CEUS follow-up
Saving (Euro)	5516.45	1640.2
Percentual saving	66.71%	37.34%

MCN 3–4 cm	ACG Clinical Guideline and European evidence-based guidelines follow-up vs CEUS follow-up	
Saving (Euro)	5162.39	
Percentual saving	58.35%	

SCN < 4 cm	European evidence-based Guidelines follow-up vs CEUS follow-up	Italian guidelines follow-up vs CEUS follow-up
Saving (Euro)	894.66	321
Percentual saving	40.73%	19.78%

For all scenarios, cost deriving from the benefit is less than the maximum amount that the decision-maker would be willing to pay for this benefit.

## Discussion

The results of our analysis suggest that a strategy based on CEUS appears to be cost-effective in managing incidental asymptomatic PCN, particularly in the surveillance of MCN/BD-IPMN with size < 1 cm. In fact, in this case it is the least expensive and appears very effective compared to “Consensus Fukuoka guidelines follow-up” and “Italian consensus follow-up”, in terms of yield in QALY gained and NMB. In case of MCN/BD-IPMN with size 1–2 cm and 2–3 cm and SCN < 4 cm, a strategy based on CEUS demonstrates a cost-effectiveness ratio not as high as we expected, although it is certainly cheaper than guidelines’ strategy: the reason of this results is the need of a more aggressive follow-up of these lesions, due to their increased risk to developing malignancies. Although the strategy of CEUS in MCN with size 3–4 cm is the most expensive, it is also more cost-effective than we expected, with an ICER below the acceptable threshold of 30,000€ per QALY gained by a health care intervention. Larger cysts possess a higher likelihood of malignancy or higher rate of progression, and their aggressive follow-up can be prohibitively costly, leading to this result.

Follow-up with CEUS yields a savings that, built over 10 years, can be substantial.

Morelli et al. report their experience regarding the surveillance of PCN with US, reducing MRI use every two years or in the case of lesion changes [42]. However, US Doppler cannot clearly evaluate the enhancement of walls and of solid components of cysts, as CEUS can do [15–17, 19].

The hypothetical analysis performed in our study found that in these scenarios, CEUS management can be a very reasonable individualized cost-effective approach.

It is important to note that our study is retrospective and based on hypothetical constructs with inherent limitations. Because there is limited published data about asymptomatic PCN, we assumed that a pancreatic cyst has already been correctly identified by CT and MRI, and the patient is completely asymptomatic. In this model we considered solitary lesions only. Multiple lesions, which are not uncommon, present more difficult management decisions, which are usually made on a case-by-case basis [5, 6, 10–14, 34, 35, 43]. The retrospective nature may also influence the cost assessment, based on the costs incurred in cohorts of patients that only underwent MRI surveillance.

In summary, the management of asymptomatic PCN presents a true challenge and we tried to establish the impact of costs on decision making. Our data and other studies show that strategies found in Literature are very effective, but costs are relatively high from a policy perspective [14, 23, 35, 38, 43].

There are also many other studies reported in Literature that affirm the role of cost effectiveness analysis in the healthcare area. Terpenning S. and colleagues review the latest studies regarding the approach to stable ischemic heart disease and suggest a better cost-effective strategy [44]. Centonze M et al. try to perform a cost-effective analysis focused on the role of Calcium Score, coronary computed tomography angiography and cardiac magnetic resonance [45]. Furthermore, according to England RW et al., using advanced imaging modalities in clinical scenarios may improve outcomes and reduce total cost of care, supporting value-based reimbursement decisions [46].

The results of our study demonstrate our strategies as the most cost/effective: adopting our simulated protocols would decrease the costs by an average of 45.52% in case of comparison with “Consensus Fukuoka guidelines follow-up” and by an average of 20.75% in case of comparison with “Consensus Italian guidelines follow-up”, yielding a nearly high QALYs and an acceptable ICER. The Consensus Fukuoka guidelines, European and Italian Guidelines serve as a template for which most providers manage this disease; our model further validates many of these recommendations. However, to this day, the extension of surveillance, the appropriate interval and type of investigations needed are not completely adapted to an individual-level [7–9, 14, 23, 35, 42].

## Conclusion

This study investigates cost-effectiveness of surveillance for each histologic type of PCN, tailoring an approach based on risk stratifications for a both safe and cost-effective management. We suggest the inclusion of CEUS as surveillance diagnostic test in asymptomatic PCNs’ follow-up, putting this improvement into a prospective long term evolution in health economics and without any presumption to replace the existing protocols. In patients with PCN and without “worrisome features” or “high risk stigmata”, abdominal CEUS could be a safe complementary approach, reducing the cost of surveillance. This model could be adapted to generate follow-up strategies for each subgroup of PCN, allowing a more efficient design.

## References

[1] Zerboni G, Signoretti M, Crippa S, Falconi M, Arcidiacono PG, Capurso G (2019). Systematic review and meta-analysis: prevalence of incidentally detected pancreatic cystic lesions in asymptomatic individuals. Pancreatology.

[2] Correa-Gallego C, Ferrone CR, Thayer SP, Wargo JA, Warshaw AL, Fernández-Del CC (2010). Incidental pancreatic cysts: Do we really know what we are watching?. Pancreatology.

[3] Jais B, Rebours V, Malleo G, Salvia R, Fontana M, Maggino L (2016). Serous cystic neoplasm of the pancreas: a multinational study of 2622 patients under the auspices of the International Association of Pancreatology and European Pancreatic Club (European Study Group on Cystic Tumors of the Pancreas). Gut.

[4] Jenssen C, Kahl S (2015). Management of Incidental Pancreatic Cystic Lesions. Viszeralmedizin.

[5] Crippa S, Fernández-del CC (2007). A selective approach to the resection of cystic lesions of the pancreas: results from 539 consecutive patients. Ann Surg.

[6] Hisada Y, Nagata N, Imbe K, Takasaki Y, Sekine K, Tajima T (2017). Natural history of intraductal papillary mucinous neoplasm and non neoplastic cyst: long-term imaging follow-up study. J Hepatobiliary Pancreat Sci.

[7] Crippa S, Fernández-Del Castillo C, Salvia R, Finkelstein D, Bassi C, Domínguez I (2010). Mucin producing neoplasms of the pancreas: An analysis of distinguishing clinical and epidemiologic characteristics. Clin Gastroenterol Hepatol.

[8] Brook OR, Beddy P, Pahade J, Couto C, Brennan I, Patel P (2016). Delayed growth in incidental pancreatic cysts: are the current American College of Radiology recommendations for follow-up appropriate?. Radiology..

[9] Crippa S, Pezzilli R, Bissolati M, Capurso G, Romano L, Brunori MP (2017). Active surveillance beyond 5 years is required for presumed branch duct intraductal papillary mucinous neoplasms undergoing non operative management. Am J Gastroenterol..

[10] Tanaka M, Fernández-Del Castillo C, Kamisawa T, Jang JY, Levy P, Ohtsuka T (2017). Revisions of international consensus Fukuoka guidelines for the management of IPMN of the pancreas. Pancreatology..

[11] Italian Association of Hospital Gastroenterologists and Endoscopists; Italian Association for the Study of the Pancreas, Buscarini E, Pezzilli R, Cannizzaro R, De Angelis C, et al. (2014) Italian consensus guidelines for the diagnostic work up and follow up of cystic pancreatic neoplasms. Dig Liver Dis. 46(6): 479–93. PMID: 24809235 Doi: 10.1016/j.dld.2013.12.019 10.1016/j.dld.2013.12.019 24809235

[12] Elta GH, Enestvedt BK, Sauer BG, Lennon AM (2018). ACG clinical guideline: diagnosis and management of pancreatic cysts. Am J Gastroenterol.

[13] European Study Group on Cystic Tumours of the Pancreas (2018) European evidence-based guidelines on pancreatic cystic neoplasms. Gut. 67(5): 789–804. PMID: 29574408 Doi: 10.1136/gutjnl-2018-316027. 10.1136/gutjnl-2018-316027 PMC5890653 29574408

[14] Das A, Ngamruengphong S, Nagendra S, Chak A (2009). Asymptomatic pancreatic cystic neoplasm: a cost-effectiveness analysis of different strategies of management. Gastrointest Endosc.

[15] D'Onofrio M, Megibow AJ, Faccioli N, Malagò R, Capelli P, Falconi M, Mucelli RP (2007). Comparison of contrast-enhanced sonography and MRI in displaying anatomic features of cystic pancreatic masses. AJR Am J Roentgenol.

[16] Faccioli N, Crippa S, Bassi C, D'Onofrio M (2009). Contrast-enhanced ultrasonography of the pancreas. Pancreatology.

[17] Beyer-Enke SA, Hocke M, Ignee A, Braden B, Dietrich CF (2010). Contrast enhanced transabdominal ultrasound in the characterisation of pancreatic lesions with cystic appearance. JOP.

[18] Kobayashi N, Sugimori K, Shimamura T, Hosono K, Watanabe S, Kato S (2012). Endoscopic ultrasonographic findings predict the risk of carcinoma in branch duct intraductal papillary mucinous neoplasms of the pancreas. Pancreatology..

[19] D'Onofrio M, Biagioli E, Gerardi C, Canestrini S, Rulli E, Crosara S, De Robertis R, Floriani I (2014). Diagnostic performance of contrast-enhanced ultrasound (CEUS) and contrast-enhanced endoscopic ultrasound (ECEUS) for the differentiation of pancreatic lesions: a systematic review and meta-analysis. Ultraschall Med..

[20] Kawada N, Uehara H, Nagata S, Tsuchishima M, Tsutsumi M, Tomita Y (2016). Mural nodule of 10 mm or larger as predictor of malignancy for intraductal papillary mucinous neoplasm of the pancreas: pathological and radiological evaluations. Pancreatol.

[21] Sidhu PS, Cantisani V, Dietrich CF, Gilja OH, Saftoiu A, Bartels E, Bertolotto M (2018). The EFSUMB guidelines and recommendations for the clinical practice of Contrast-Enhanced Ultrasound (CEUS) in non-hepatic applications: update 2017 (Long Version). Ultraschall Med.

[22] Faccioli N, D'Onofrio M, Comai A, Cugini C (2007). Contrast-enhanced ultrasonography in the characterization of benign focal liver lesions: activity-based cost analysis. Radiol Med.

[23] Faccioli N, Dietrich CF, Foti G, Santi E, Comai A, D'Onofrio M (2019). Activity-based cost analysis of including contrast-enhanced ultrasound (CEUS) in the diagnostic pathway of focal pancreatic lesions detected by abdominal ultrasound. Ultraschall Med.

[24] Sanders GD, Neumann PJ, Basu A, Brock DW, Feeny D, Krahn M (2016). Recommendations for conduct, methodological practices, and reporting of cost-effectiveness analyses: second panel on cost-effectiveness in health and medicine. JAMA..

[25] Cohen DJ, Reynolds MR (2011). Interpreting the results of cost-effectiveness studies. J Am Coll Cardiol.

[26] Eurostat, [database online] Statistical Office of the European Communities, Labour market statistics. Luxembourg. Issue number 48/2012. Doi: 10.2785/15405

[27] Neumann PJ, Cohen JT, Weinstein MC (2014). Updating cost-effectiveness—the curious resilience of the $50,000-per-QALY threshold. N Engl J Med..

[28] OECD/EU (2018) Health at a Glance: Europe 2018: State of Health in the EU Cycle, OECD Publishing, Paris/EU, Brussels

[29] Caraiani C, Dong Y, Rudd AG, Dietrich CF (2018). Reasons for inadequate or incomplete imaging techniques. Med Ultrason.

[30] Gruppo di lavoro misto SIRM-SNR (2006) Metodologia di determinazione dei volumi di attività e della produttività dei medici radiologi. Omicron Ed (Genova)

[31] Buscaglia JM, Giday SA, Kantsevoy SV, Jagannath SB, Magno P, Wolfgang CL (2009). Patient- and cyst-related factors for improved prediction of malignancy within cystic lesions of the pancreas. Pancreatology..

[32] Tanno S, Nakano Y, Sugiyama Y, Nakamura K, Sasajima J, Koizumi K (2010). Incidence of synchronous and metachronous pancreatic carcinoma in 168 patients with branch duct intraductal papillary mucinous neoplasm. Pancreatology..

[33] Crippa S, Capurso G, Cammà C, Fave GD, Castillo CF, Falconi M (2016). Risk of pancreatic malignancy and mortality in branch-duct IPMNs undergoing surveillance: a systematic review and meta-analysis. Dig Liver Dis.

[34] de Pretis N, Mukewar S, Aryal-Khanal A, Bi Y, Takahashi N, Chari S (2017). Pancreatic cysts: diagnostic accuracy and risk of inappropriate resections. Pancreatology.

[35] Huang ES, Gazelle GS, Hur C (2010). Consensus guidelines in the management of branch duct intraductal papillary mucinous neoplasm: a cost-effectiveness analysis. Dig Dis Sci.

[36] Faccioli N, Foti G, Casagranda G, Santi E, D'Onofrio M (2018). CEUS versus CT Angiography in the follow-up of abdominal aortic endoprostheses: diagnostic accuracy and activity-based cost analysis. Radiol Med.

[37] Best LM, Rawji V, Pereira SP, Davidson BR, Gurusamy KS (2017). Imaging modalities for characterising focal pancreatic lesions. Cochrane Database Syst Rev.

[38] Weinberg BM, Spiegel BM, Tomlinson JS, Farrell JJ (2010). Asymptomatic pancreatic cystic neoplasms: maximizing survival and quality of life using Markov-based clinical nomograms. Gastroenterology.

[39] van der Gaag NA, Berkhemer OA, Sprangers MA, Busch OR, Bruno MJ, de Castro SM (2014). Quality of life and functional outcome after resection of pancreatic cystic neoplasm. Pancreas.

[40] Lien K, Tam VC, Ko YJ, Mittmann N, Cheung MC, Chan KK (2015). Impact of country-specific EQ-5D-3L tariffs on the economic value of systemic therapies used in the treatment of metastatic pancreatic cancer. Curr Oncol.

[41] Fitzsimmons D, Kahl S, Butturini G, van Wyk M, Bornman P, Bassi C (2005). Symptoms and quality of life in chronic pancreatitis assessed by structured interview and the EORTC QLQ-C30 and QLQ-PAN26. Am J Gastroenterol..

[42] Morelli L, Guadagni S, Borrelli V, Pisano R, Di Franco G, Palmeri M (2019). Role of abdominal ultrasound for the surveillance follow-up of pancreatic cystic neoplasms: a cost-effective safe alternative to the routine use of magnetic resonance imaging. World J Gastroenterol.

[43] Rosenkrantz AB, Xue X, Gyftopoulos S, Kim DC, Nicola GN (2018). Downstream costs associated with incidental pancreatic cysts detected at MRI. AJR Am J Roentgenol.

[44] Terpenning S, Stillman A (2020) Cost-effectiveness for imaging stable ischemic disease. Br J Radiol 93(1113): 20190764. Doi: 10.1259/bjr.20190764. Epub 2020 Apr 23. PMID: 32302209; PMCID: PMC7465859 10.1259/bjr.20190764 PMC7465859 32302209

[45] Centonze M, Steidler S, Casagranda G, Alfonsi U, Spagnolli F, Rozzanigo U (2020). Cardiac-CT and cardiac-MR cost-effectiveness: a literature review. Radiol Med.

[46] England RW, Sheikhbahaei S, Solomon AJ, Arbab-Zadeh A, Solnes LB, Bronner J (2021). When more is better: underused advanced imaging exams that can improve outcomes and reduce cost of care. Am J Med.

